# Preclinical evaluation of a clinical prototype transrectal diffuse optical tomography system for monitoring photothermal therapy of focal prostate cancer

**DOI:** 10.1117/1.JBO.27.2.026001

**Published:** 2022-02-01

**Authors:** Celina L. Li, Carl J. Fisher, Brian C. Wilson, Robert A. Weersink

**Affiliations:** a University of Toronto, Department of Medical Biophysics, Toronto, Canada; b University Health Network, Princess Margaret Cancer Centre, Toronto, Canada; c University of Toronto, Department of Radiation Oncology, Toronto, Canada; d University of Toronto, Institute of Biomedical Engineering, Toronto, Canada

**Keywords:** transrectal diffuse optical tomography, prostate cancer, photothermal therapy, image reconstruction, treatment monitoring

## Abstract

**Significance:**

Our work demonstrates in preclinical models that continuous-wave transrectal diffuse optical tomography (TRDOT) can be used to accurately monitor photothermal therapy (PTT) and, in particular, the progression of the photocoagulation boundary toward the rectum. When used in patients, this should prevent rectal damage during PTT, thereby achieving maximum treatment efficacy while ensuring safety, using a technology platform suitable for wide dissemination.

**Aim:**

We aim to validate that TRDOT measurements analyzed using a shape-based image-reconstruction algorithm (SBDOT) allow localization of the photocoagulation boundary during PTT within ±1  mm toward the rectum in the transverse plane.

**Approach:**

TRDOT measurements were performed in tissue-simulating phantoms, *ex vivo* tissues, and an *in vivo* canine prostate model. The accuracy and sensitivity of reconstructing the size and location of the coagulation zone were determined, based on changes in the tissue absorption and reduced scattering coefficients upon photocoagulation. The reconstruction also yields the native and coagulated tissue optical properties.

**Results:**

The TRDOT measurements and SBDOT reconstruction algorithm were confirmed to perform sufficiently well for clinical translation in PTT monitoring, recovering the location of the coagulation boundary within ±1  mm compared to the true value as determined by direct visualization postexcision and/or MRI.

**Conclusions:**

Implementing previously described TRDOT instrumentation and SBDOT image reconstruction in different tissue models confirms the potential for clinincal translation, including required refinements of the system and reconstruction algorithm.

## 1 Introduction

Screening programs for prostate cancer (PCa) have increased the detection rate of low- and intermediate-risk disease confined to the prostate (focal tumors), for which treatment options range from active surveillance, with risk of disease progression, to radical therapies (prostatectomy and radiotherapy) that carry significant rates of urinary incontinence and erectile dysfunction.^1^^,^^2^ Focal ablative therapies, in which treatment is targeted to the dominant (index) lesion rather than the whole gland, aim to minimize morbidity while achieving successful cancer control and preserving the possibility of retreatment. Several focal modalities, including high-intensity focused ultrasound, cryotherapy, irreversible electroporation, and photodynamic therapy, have shown promising results with short- to medium-term follow up.^3^^–^^8^

A further option is photothermal therapy (PTT), in which near-infrared (NIR) light is delivered via interstitial optical fibers to destroy the target index lesion by thermal coagulation (>∼55°C).^9^^–^^12^ The advantages include good spatial control from accurate fiber insertion and selective tumor destruction regulated by light distribution depth and delivered energy. Photothermal treatment of focal PCa is usually monitored by magnetic resonance image-based thermometry (MRT), providing online feedback of thermal dose as an indirect indicator of the growth of the coagulation zone. Several clinical trials and case studies in low/intermediate-risk PCa patients have demonstrated that PTT can achieve minimal adverse effects and biopsy-confirmed tumor ablation in most cases, with 70% to 80% being radiologically and pathologically disease-free in the targeted ablation zone at 3 to 18 months follow up.^13^^–^^18^ However, due to the limited spatial resolution and indirect monitoring of the coagulation zone based on MRI temperature mapping, the photocoagulation front (boundary of thermal coagulation) cannot be delineated precisely enough to achieve complete tumor destruction in all cases, particularly for tumors in the posterior capsule, because of the need to prevent damage to the rectal wall. In one recent study, for example, only 11 out of 23 patients had complete target ablation.^19^

**Fig. 1:**
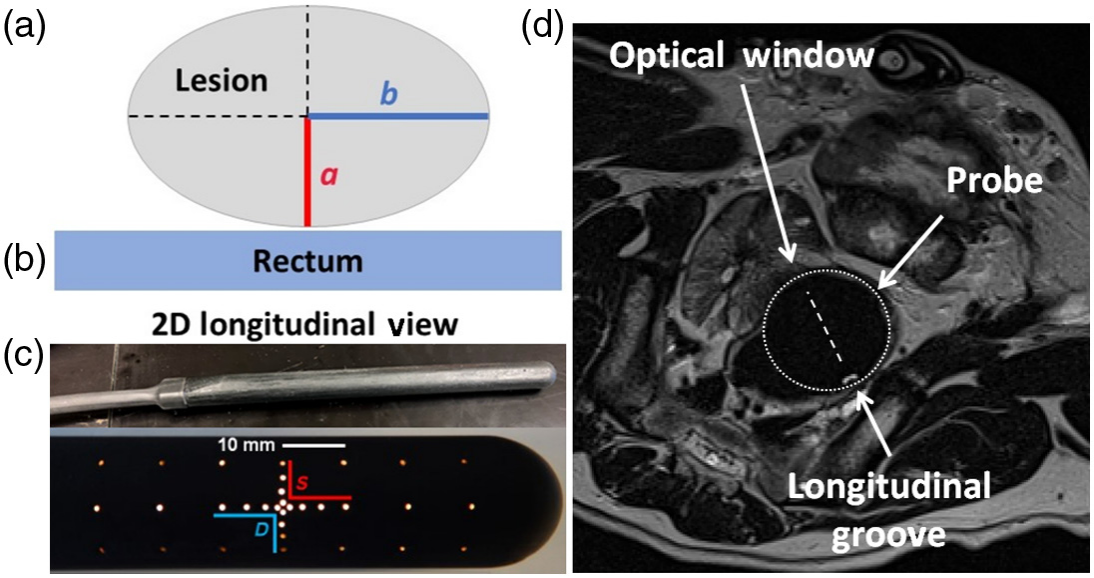
Transrectal DOT probe: (a) longitudinal view showing the geometry of the photothermal coagulation zone relative to the rectum and indicating the transverse (a) and longitudinal (b) radii. (b) Photograph of the probe and (c) arrangement of source (S) and detector (D) fibers within the optical window. (d) MR image (transverse plane) of the probe in canine rectum: the longitudinal orientation groove is visible.

Diffuse optical tomography (DOT) uses visible/NIR light to sample a tissue volume of interest, allowing quantitative mapping of the optical absorption ($\mu_{a}$) and reduced scattering ($\mu_{s}^{\prime }$) coefficients that carry morphological and functional information.^20^ DOT can be used stand alone or in combination with radiological imaging.^21^ Transrectal DOT (TRDOT) has also been investigated for prostate tumor imaging with transrectal ultrasound (TRUS) to enhance spatially accuracy.^22^^–^^25^ We have previously reported the design, construction, and calibration of a prototype continuous-wave DOT system and transrectal probe. Preliminary tests demonstrated that TRDOT signals are sensitive to changes in tissue absorption and scattering due to PTT^26^^–^^28^ and had sufficient dynamic range. The probe is shown in Fig. 1.

We also previously reported a shape-based algorithm (SBDOT) to reconstruct images of the treatment zone,^29^ based on assuming an approximately ellipsoidal, sharp, and monotonically expanding boundary between the coagulated and native tissue. Computer simulations^26^ and preliminary phantom experiments^27^^,^^28^ suggested that it is possible to localize the coagulation boundary within ±1  mm in the transverse plane, which we had set as the target accuracy to avoid rectal damage while allowing maximal tumor ablation. We demonstrated that a twofold increase in scattering upon coagulation is required for reconstruction accuracy.^26^^,^^29^

The primary objective of the current work is then to validate these prelimary findings systematically and in depth, including under *in vivo* conditions, as a critical step toward clinical translation. Hence, we first validated the SBDOT algorithm in tissue-simulating phantoms to reconstruct zones of fixed diameter and location and with known optical properties representative of coagulated tissue. DOT monitoring of the coagulation zone during PTT was then tested in *ex vivo* tissues under well-controlled conditions using a simulated transrectal geometry. This allowed detailed quantitative comparisons between the reconstructed photocoagulation zone compared to simultaneous MR thermometry as treatment progressed. Finally, the system and reconstructions were validated under clinically relevant *in vivo* conditions during photothermal treatment of normal prostate in a canine model with the TRDOT probe placed in the rectum. These studies critically inform planned first clinical trials of this treatment monitoring technology.

## 2 Materials and Methods

All canine procedures were approved by the Animal Care Committee, University Health Network, Toronto, Canada and complied with regulations of the Canadian Council on Animal Care.

### 2.1 Instrumentation and Data Acquisition

Details of the measurement and reconstruction workflow are described in the Supplementary Material. The system currently utilizes three wavelengths: 670, 750, and 808 nm corresponding, respectively, to the main absorption peak of lipid-porphyrin nanoparticles under development for thermal dose enhancement during PTT,^30^ an absorption peak of deoxygenated hemoglobin (Hb), and the $\mathrm{Hb}\text{-}{\mathrm{HbO}}_{2}$ isosbestic point. The MRI-compatible transrectal probe is cylindrical (diameter 25 mm) with 400  μm optical fibers arranged within an area (20 mm longitudinally × 16 mm transversally) centred 5 cm from the probe tip. The eight light delivery and eight collection fibers exit the probe perpendicular to and flush with the surface, as seen in Fig. 1. Powers of 20, 9, and 5.5 mW were used for the 670-, 750-, and 808-nm lasers, respectively. Grooves in the longitudinal and the transversal planes enable MRI positioning and orientation. Phantom measurements were made at 670, 750, and 808 nm, whereas *ex viv*o and *in vivo* measurements were made only at 750 nm to minimize the sampling time in following the dynamic coagulation changes: 28 s per scan using 8 sources when using 8 sources. Measurements in a reference phantom of known optical properties provide calibration factors for each source–detector pair,^28^ repeated for each measurement session.

### 2.2 Phantom Study

**Fig. 2:**
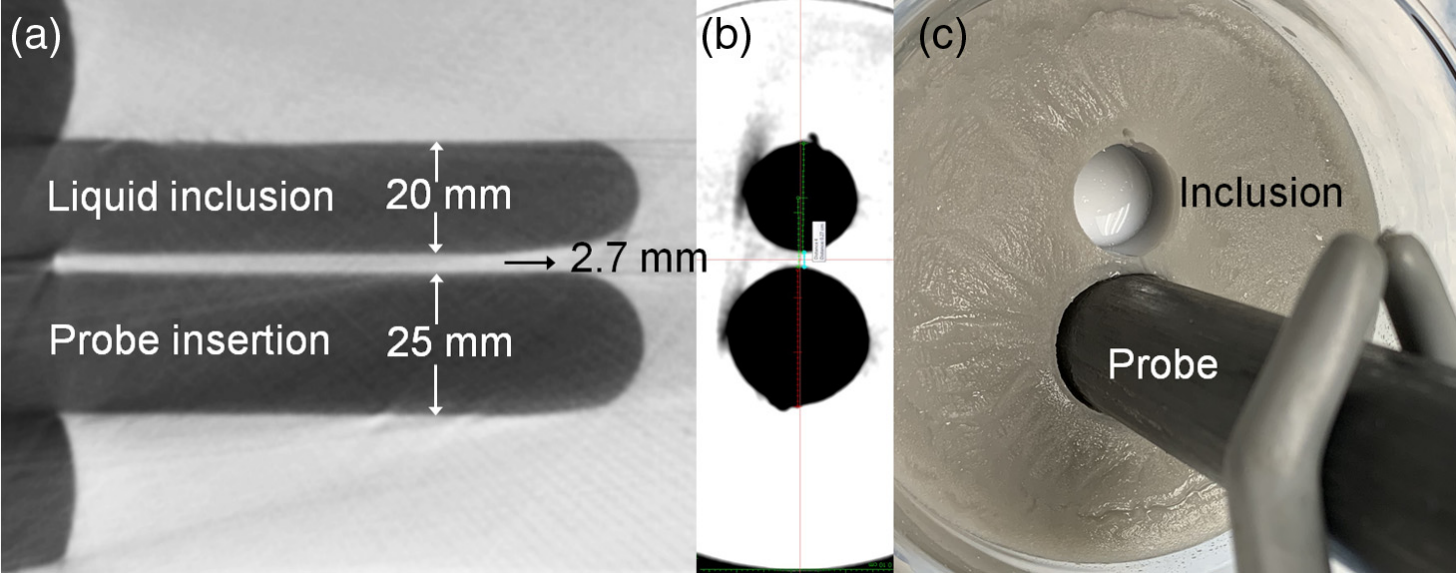
Agar phantom with inclusion simulating the photocoagulation zone and holding the probe. Cone-beam CT images in (a) coronal and (b) axial views ($0.39\times 0.39\times 0.39\;{\mathrm{mm}}^{3}\text{ voxels}$). (c) Top-view photograph.

Phantom mixtures were prepared using laboratory-grade agar (BioShop Canada Inc., Burlington, Ontario, Canada) at 2% weight/volume as the bulk matrix, Intralipid^®^ 20% stock solution (Baxter, Mississauga, Ontario, Canada) for optical scattering, India Ink for optical absorber, and 5% w/v OMNIPAQUE 350 iohexol injection USP (GE Healthcare, Mississauga, Ontario, Canada) as a CT contrast agent and 0.8% NaCl as an antibacterial agent. A previously validated spatially resolved flat-top diffuse reflectance (DR) probe was used to measure the optical properties^31^ in a 6-cm diameter, 2.5-cm height phantom block. Separately, two cylindrical wells were fabricated in a 1000-ml container, one of 20-mm diameter representing the coagulated zone with altered optical properties and one of 25-mm diameter for the probe. Each well was 100-mm deep with an edge-to-edge separation of 2.7 mm. The sizes and separation of the wells were confirmed by cone-beam CT imaging: Fig. 2(a). The 20-mm well was filled to a height of ∼90  mm using six different concentrations of Intralipid and India Ink to represent zones of altered, i.e., coagulated, optical properties. The optical properties of each liquid inclusion and of the agar matrix were measured at 670, 750, and 808 nm using the DR probe that was independently calibrated in a commercial solid reference phantom (Biomimic™, INO, Quebec, Canada).^28^

The probe well was water filled for optical coupling. All measurements were repeated 5 times with the phantom covered with blackout fabric and were corrected for dark counts [Fig. 2(b)]. Reconstructions were performed using different initial estimates of the inclusion radius (6, 8, 10, and 12 mm) and length (10 and 45 mm) combined with different regularization values. Estimated errors in the reconstructed optical properties and shape parameters were calculated using the standard methods for nonlinear regressions based on calculating the Hessian matrix. The optical properties of the simulated coagulation zone and the agar phantom as measured by the DR probe were used as initial values in reconstruction and we also examined the effect of varying the initial $\mu_{a}$ and $\mu_{s}^{\prime }$ values, both individually and in combination, using the methodology shown in detail in Fig. S2 in the Supplementary Material. The reconstructions were based on NIRFAST^32^ meshing and were performed in MATLAB (The MathWorks, Inc., Natick, Massachusetts, USA). CT images were analyzed using ImageJ (National Institutes of Health, Bethesda, Maryland, USA).

### 2.3 *Ex Vivo* Study

TRDOT monitoring of photocoagulation was performed in *ex vivo* bovine and porcine muscle using a setup similar to the prior preliminary experiments.^28^ Briefly, two 8- to 12-mm-thick tissue slabs of either bovine or porcine muscle were used. The probe was fixed with the optical window facing upward on the table of a 1.5-T MRI system (Aera: Siemens, Toronto, Ontario, Canada). A first tissue slab was placed on top in close contact. A 1.7-mm diameter, 15-mm long diffusing-tip PTT treatment fiber delivering 5 W at 980 nm was placed on the tissue parallel to the long axis of the probe and centered on the optical window. The fiber and probe positions were confirmed by a fast MRI localization scan. $T_{2}w$ Turbo Spin Echo scans then were acquired pre- and post-PTT to image the photocoagulation zone in the axial plane (voxel size: $1\times 1\times 3\;{\mathrm{mm}}^{3}$; TR: 3600 ms; and TE: 57 ms). Repeated MRT scans (GRE; TR: 46 ms; TE: 9 ms; $1\times 1\times 3\;{\mathrm{mm}}^{3}\text{ voxels}$) and 750 nm DOT scans were acquired simultaneously for ∼140  s immediately prior to PTT, ∼700  s during treatment delivery, and ∼300 to 400 s during tissue cool down. The tissue slabs were then separated and the final photocoagulation diameter was measured using a ruler in the longitudinal and cross-sectional directions.

Rather than a reference phantom, the native tissue optical properties, either measured using the DR probe or from the literature were used for TRDOT calibration, to minimize any effect of tissue–probe contact. These properties were input to a forward model of the TRDOT signals, which were compared to the measured signals to calculate individual source–detector calibration factors. The transverse and longitudinal radii and the optical properties of the coagulation zone were reconstructed using the SBDOT algorithm. For comparison, following 3×3 Gaussian smoothing, the 55°C contour from the MRT images was taken as the photocoagulation boundary at each treatment time point, noting in particular the distance between the coagulation boundary and the probe. The PTT fiber position and final photocoagulation boundary were also estimated from the posttreatment T2w MR images. As detailed in the Supplementary Material S2, a range of initial optical properties of the coagulation tissue were used in the SBDOT reconstructions to test the sensitivity to these starting values.

### 2.4 *In Vivo* Study

**Fig. 3:**
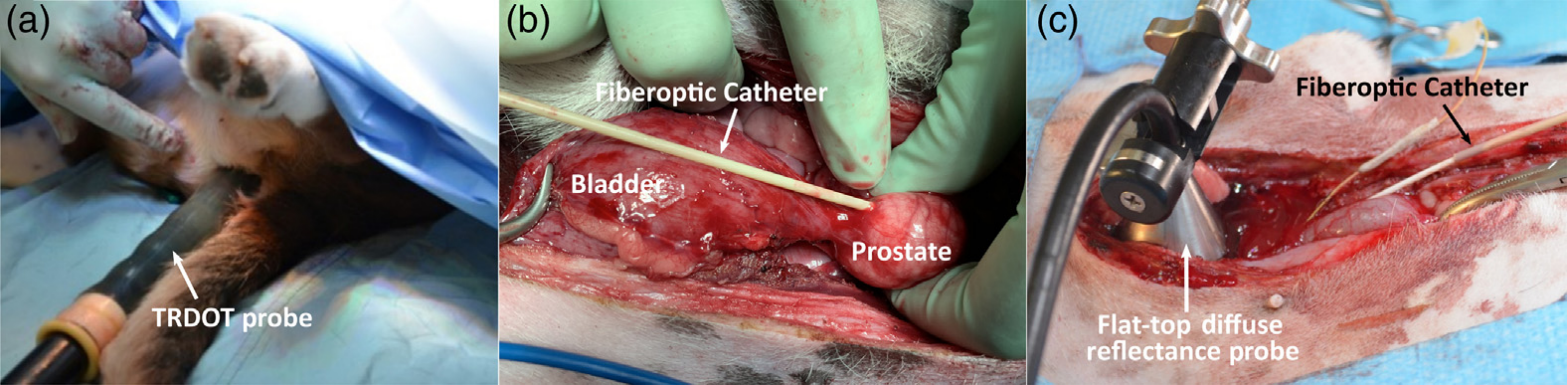
*In vivo* setup showing (a) insertion of the treatment fiber at laparotomy; (b) the TRDOT probe placed in the rectum with the optical window facing the center of prostate; and (c) the flat-top DR probe held rigidly on the ventral surface of the exposed prostate.

*In vivo* PTT of the canine prostate, which is similar in size to the adult human prostate, was performed in 6 male beagle dogs (12- to 14-month old, 12 to 14 kg weight: Marshall Bioresources Inc., NY, USA). All procedures were performed under general anesthesia following 12 h fasting and 3 warm-water enemas. Laparotomy, as required for a different concurrent study, allowed direct access to the prostate. For the laparotomy, an incision was made from the umbilicus to the pubis, exposing the bladder, prostate, and kidney. The bladder was retracted toward the umbilicus and sutured to the abdominal wall to minimize free-organ motion, which also exposed and stabilized the prostate. The PTT treatment fiber was inserted through a 14-G cannula/introducer to a depth of ∼1  cm [see Fig. 3(a)].

The TRDOT probe was inserted into the rectum in a sterile condom with lubricant gel and aligned with the prostate [Fig. 3(b)] by palpation. The optical properties of the untreated prostate at 750 nm were measured using the DR probe in direct contact with the anterior prostate surface via the laparotomy [Fig. 3(c)]. All TRDOT scans were performed at 750 nm, starting immediately prior to PTT. The first half of the treatment was given using a laser power of 3 W, which was increased to 5 W at the midpoint. This protocol minimized blood coagulation at the fiber tip while maximizing the coagulation zone. It also tested the ability of TRDOT to track the growth rate of the coagulation zone. Two different TRDOT monitoring protocols were evaluated: (i) PTT over 744 s, with TRDOT monitoring using 8 light sources (28 s per complete scan) and (ii) PTT for 210 s and TRDOT using only 4 sources to reduce the acquisition time to 14 s. In both cases, TRDOT monitoring continued for ∼100  s following the end of treatment.

The SBDOT algorithm was used to reconstruct the expanding photocoagulation zone and to measure the pre- and post-PTT optical properties. This utilized a NIRFAST mesh comprising four regions: photocoagulation zone, untreated prostate, rectum, and surrounding tissues. TRDOT data were calibrated as per the *ex vivo* study.

## 3 Results and Discussion

### 3.1 Phantom Study

**Fig. 4:**
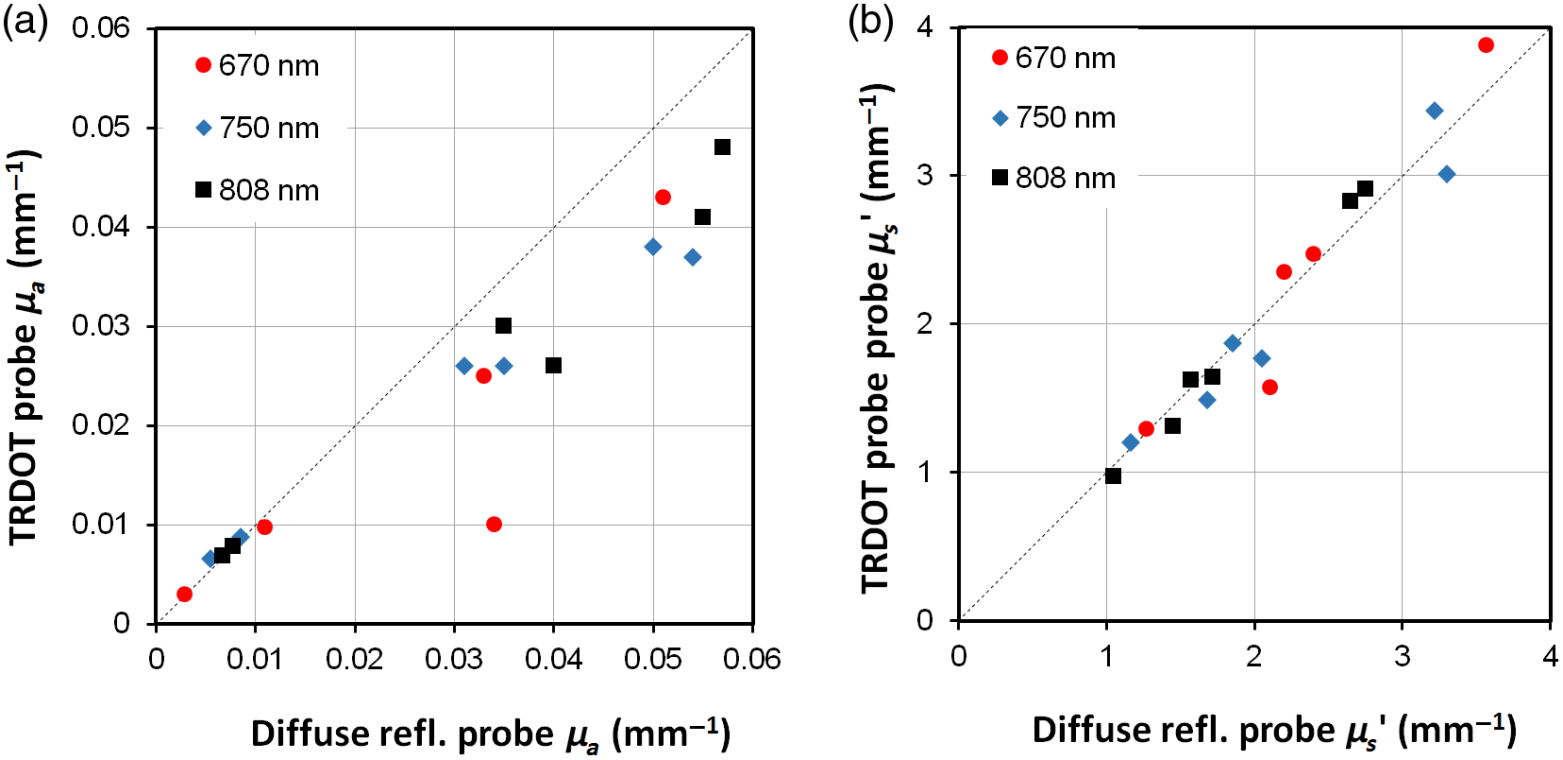
Comparison of (a) $\mu_{a}$ and (b) $\mu_{s}^{\prime }$ of the liquid inclusions measured by the TRDOT and flat-top DR probes. The signal was below the measurement limit at 670 nm with the highest absorption. Lines of equality are indicated.

The $\mu_{a}$ and $\mu_{s}^{\prime }$ values of the agar phantom were 0.009 and $1.08\;{\mathrm{mm}}^{-1}$, 0.006 and $1.07\;{\mathrm{mm}}^{-1}$, and 0.004 and $1.01\;{\mathrm{mm}}^{-1}$ at 670, 750, and 808 nm, respectively, with coefficients of variation of 9.8% for $\mu_{a}$ and 4.0% for $\mu_{s}^{\prime }$ (n=10). The optical properties of the liquid inclusions simulating the coagulation zone as calculated from the TRDOT measurements are plotted against the reference DR probe measurements in Fig. 4. The SBDOT reconstructed radius was r=10.8±0.2  mm (where the error represents the average across measurements at the three wavelengths), compared with the true value of 10 mm. Table 1 summarizes the average reconstructed values at each wavelength.

**Table 1:** Inclusion optical properties showing the target range of $\mu_{a}$ and $\mu_{s}^{\prime }$, the measured range over 670, 750, and 808 nm using the DR probe and the TRDOT probe.

	$\mu_{a}$ (${\mathrm{mm}}^{-1}$)			$\mu_{s}^{\prime }$ (${\mathrm{mm}}^{-1}$)		
	Target	DR	TRDOT	Target	DR	TRDOT
Inclusion 1	0.007 to 0.010	0.007 to 0.011	0.008 to 0.01	1.05 to 1.30	1.05 to 1.27	1.0 to 1.3
Inclusion 2	0.007 to 0.010	0.003 to 0.007	0.003 to 0.007	1.85 to 2.40	1.7 to 2.4	1.64 to 2.47
Inclusion 3	0.03 to 0.036	0.034 to 0.04	0.01 to 0.026	1.85 to 2.40	1.45 to 2.1	1.31 to 1.57
Inclusion 4	0.05 to 0.06	0.051 to 0.055	0.038 to 0.043	1.85 to 2.40	1.6 to 2.2	1.62 to 2.35
Inclusion 5	0.03 to 0.036	0.031 to 0.035	0.025 to 0.03	2.75 to 3.50	2.7 to 3.6	2.83 to 3.88
Inclusion 6^a^	0.05 to 0.06	0.054 to 0.057	0.037 to 0.048	2.75 to 3.50	2.9 to 3.4	2.91 to 3.44

a Measurements for inclusion 6 do not include data at 670 nm due to high-signal attenuation affecting recovery of optical properties.

TRDOT recovered the scattering coefficients within ±3% but systematically underestimated the absorption by about 20%. The impact of errors in reconstructed optical properties on the reconstructed size was assessed by fixing the optical properties over a range from −100% to +300% of the true values, whereas the scattering coefficient was varied from −100% to 200%. Variations in $\mu_{a}$ of up to ±30% and in $\mu_{s}^{\prime }$ of up to ±40% caused <0.7  mm variations in the reconstructed inclusion radius. The reconstructed longitudinal size converged to the maximum dimension (20 mm) of the optical window, which was shorter than the actual value of 45 mm. However, this was as expected and the window is long enough to encompass most clinical cases, in which the diffusing optical fibers for treatment light delivery rarely exceed 30 mm.

Generally, the reconstructions were insensitive to varying the initial input values for the optical properties and lesion size. For example, varying the initial estimate of the transverse lesion radius between 2 and 12 mm gave a coefficient of variation of only ∼0.2  mm in radius across 21 samples. When the initial optical properties were varied by −40% to +80% of the true values, the reconstructed radius was 10.5±0.6  mm averaged over all reconstructions (n=99) from the 670-, 750-, and 808-nm data. Only when these initial estimates were varied by more than +100% or -40% did the reconstructed transverse radius deviate from the true values by more than 2 mm (Fig. S3 in the Supplementary Material). Published optical properties of human *in vivo* prostate range widely,^33^^–^^36^ from 0.01 to $0.16\;{\mathrm{mm}}^{-1}$ for $\mu_{a}$ and from 0.1 to $4\;{\mathrm{mm}}^{-1}$ for $\mu_{s}^{\prime }$ between 730 and 790 nm, depending on measurement technique. The effective attenuation coefficient $\mu_{\mathrm{eff}}$ is more consistent at between 0.20 and $0.33\;{\mathrm{mm}}^{-1}$, and within a patient group has a lower standard deviation than $\mu_{a}$ and $\mu_{s}^{\prime }$.^34^ If the primary source of these variations is technical rather than biological, then the confidence interval for the starting optical properties should converge as the clinical data using TRDOT during PTT increases, thereby further improving reconstruction of the coagulation zone boundary.

### 3.2 *Ex Vivo* Study

Table 2 summarizes the initial (“native”) optical properties of each tissue used for the reconstructions as well as the coagulated tissues. Due to low absorption and scattering of porcine muscle, the native optical properties were not accurately recovered using the DR probe, so that the initial estimates were also extrapolated from literature values at 600 nm^37^ and 750 nm.^38^^–^^43^

**Table 2:** Optical properties of native and coagulated tissues at 750 nm measured with the TRDOT and fat-top DR probes, and compared to the literature values.^37^^–^^43^

	Optical property (${\mathrm{mm}}^{-1}$)	Native		Coagulated		
		DR	Literature	TRDOT	DR	Literature
Porcine	$\mu_{a}$		0.01 to 0.025	0.072 ± 0.007	0.036 ± 0.005	0.10
	$\mu_{s}^{\prime }$		0.3 to 0.4	1.4 ± 0.4	1.5 ± 0.1	3.0
Bovine	$\mu_{a}$	0.0040 ± 0.0005	0.01 to 0.03	0.062 ± 0.005	0.028 ± 0.007	0.03 to 0.04
	$\mu_{s}^{\prime }$	0.87 ± 0.01	0.34 to 0.70	2.3 ± 0.3	2.3 ± 0.3	1.5 to 2

For the coagulated tissues, the measured scattering coefficients for bovine muscle using either the DR or TRDOT probes are consistent with literature values, while those for porcine tissue are ∼50% of the literature value. The absorption coefficients vary markedly between the DR probe, TRDOT probe, and literature values. Most likely this reflects differences in blood content due to tissue collection and handling, as could be resolved by measuring at wavelengths above and below the Hb isosbestic point.

**Fig. 5:**
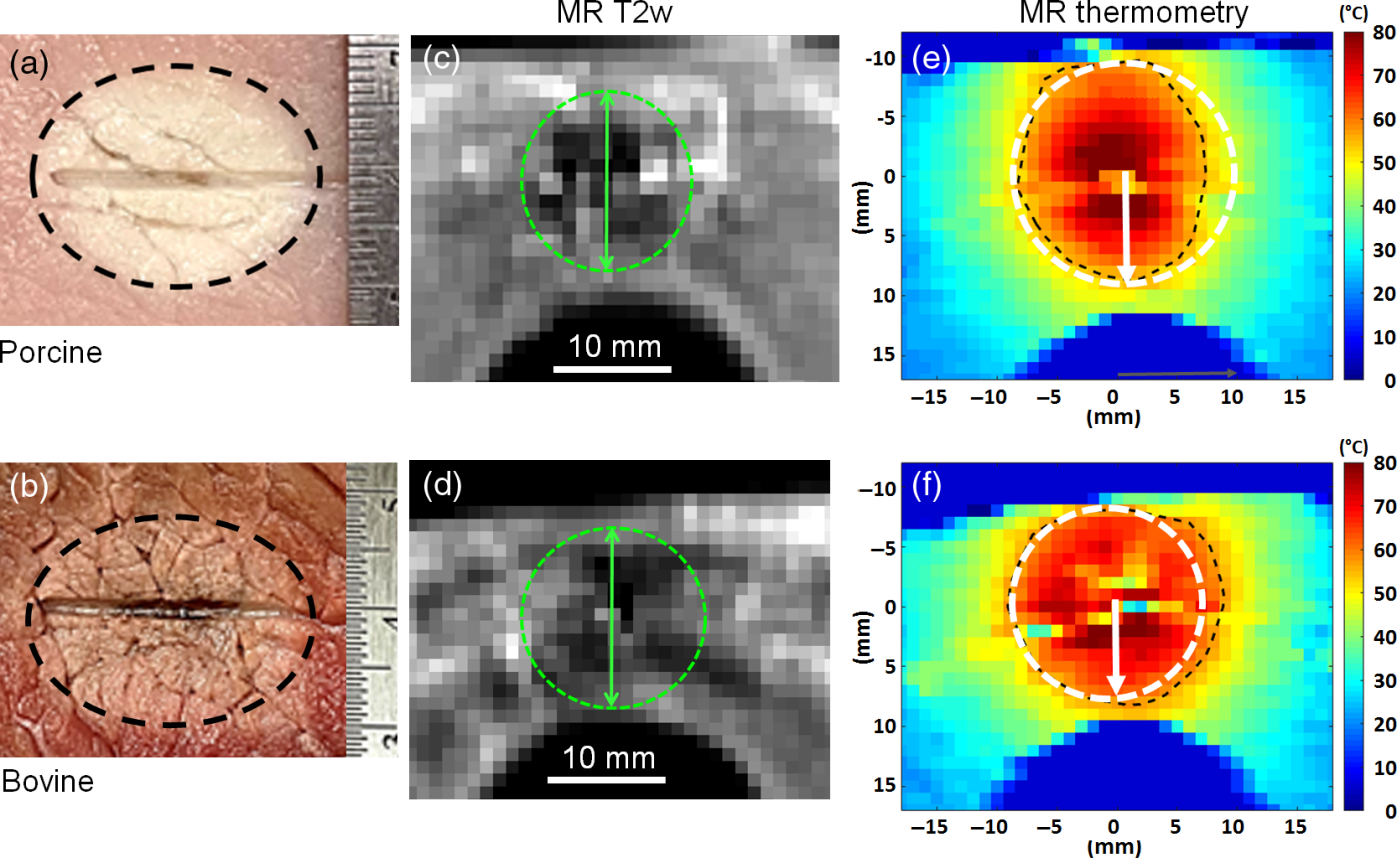
Examples of post-PTT coagulation zones in *ex vivo* tissues: (top) porcine and (bottom) bovine. (a), (b) Photographs at 15 min post-PTT in the coronal plane with TRDOT boundary shown as dashed black lines. (c), (d) Transverse MR $T_{2}w$ images with the lesion boundary outlined in green. (e), (f) False-color MRT images showing the 55°C temperature contours (black dashed line) immediately at the end of PTT. White dashed circles represent the reconstructed TRDOT boundaries centered on the treatment fiber.

Figure 5 shows photographs of the coagulation zone, each at 15 min post-PTT, MRI $T_{2}w$ images overlaid with an estimate of coagulation zone boundary based on contrast within each image, as well as MRT images taken immediately at the end of treatment, with the 55°C contour shown as a black dashed line and the SBDOT lesion shown as a white dashed line. The photocoagulation zones are clearly visible on the photographys and T2w images.

**Fig. 6:**
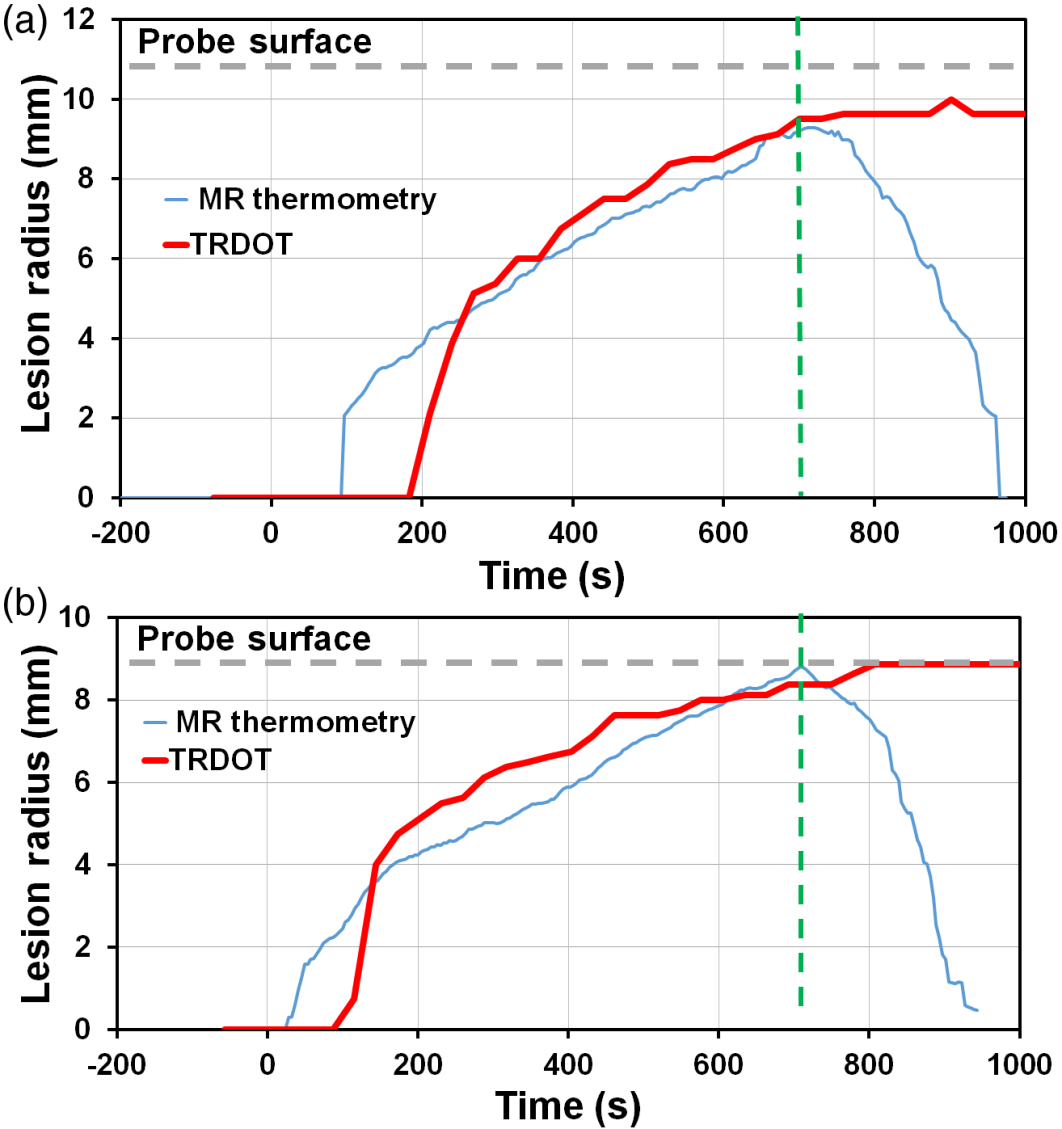
Comparison in *ex vivo* (a) porcine and (b) bovine muscle of the coagulation radius given by the 55°C MRT contour and from the SBDOT reconstruction. PTT started at t=0  s and stopped t=700  s (dashed green line).

In Fig. 6, the temporal evolution of the coagulation zone size in the two tissues from the SBDOT reconstruction is compared with the 55°C MRI contours, whereas Table 3 summarizes the final lesion sizes. Note that the temperature starts to fall as soon as the treatment laser is turned off, whereas the reconstructed coagulation zone remains constant, as expected. In general, SBDOT systematically overestimates the lesion size compared with MRT in the earlier stages of treatment when the coagulation zones were small, albeit by <1  mm. However, the two monitoring methods came into close agreement as the coagulation zone expanded and as the coagulation boundary progressed toward the transrectal probe. This was confirmed also by the direct photographic measurements at the end of treatment.

The accuracy in the coagulation size derived from MRT is limited by the axial pixel resolution ($1\times 1\;{\mathrm{mm}}^{2}$) and uncertainty in the temperature assumed for coagulation: the transverse radius increases by up to 0.6 mm as coagulation thresholds were increased from 53°C to 57°C. The accuracy of the TRDOT reconstructions was estimated by the following bootstrap procedure. For each measurement set, i.e., at each treatment time point, the reconstruction was repeated 50 times using random sets of the 64 source–detector pairs^44^ and the standard deviation in the coagulation size was calculated. The overall uncertainty in the reconstructed coagulation zone radius was ±0.6  mm transversely and ±3  mm longitudinally. This could not be attributed to instrument noise, which was <1% for all source–detector pairs, both at baseline and during PTT monitoring.

**Table 3:** PTT lesion radius (mm) at the end of treatment from T2w MRI and MR thermometry (55°C threshold), TRDOT, and direct measurement from photographs.

Tissue	PTT fiber depth	Transverse (mm)			Longitudinal (mm)		
		TRDOT	MR T2w	MRT	TRDOT	MR T2w	Photo
Porcine	10.7	9.6	7.3	9.1	12.0	N/A	12.5
Bovine	9.0	9.0	8.0	9.0	11.0	10.9	12.5

Note: N/A, not available.

Since the TRDOT probe operates in reflectance geometry, it is less sensitive to optical changes occurring deep in the tissue. Consequently, early in the treatment, the changes in detected optical signal are small, resulting in an underestimate of the size of the coagulation zone. However, this is not of concern in this clinical application. If required, it could be addressed by placing interstitial source/detector fibers within the treatment zone but at the cost of added complexity and invasiveness. It is also possible that our light modeling method is suboptimal, especially at the boundary of the native and coagulated tissues. We are investigating the mesh resolution and assumption of diffusion theory by comparisons to Monte Carlo simulations.

Reconstruction of the final coagulation lesion size was generally insensitive to the initial estimates of the optical properties during reconstruction: varying the initial $\mu_{a}$ and $\mu_{s}^{\prime }$ values by a factor of 2 typically resulted in <10% error in the reconstructed radius.

### 3.3 *In Vivo* Study

Table 4 summarizes the optical properties of native prostate tissue *in vivo* at 750 nm measured by the DR and the TRDOT probes, those of coagulated prostate measured by the TRDOT probe and, for comparison, literature values of *ex vivo* samples measured using a double integrating sphere method.^45^ The DR values were used as the initial parameters in the reconstruction algorithm. The $\mu_{a}$ values of native prostate measured by the two probes were lower than the published *ex vivo* canine prostate values at 1064 and 633 nm.^45^ The absorption decreased by about 40% upon photocoagulation. For $\mu_{s}^{\prime }$ in native prostate, the DR measurement was almost twice the TRDOT value, likely due to the former measuring only anterior prostate and the latter having contributions from 2 to 3 mm of rectal wall and connective tissue in addition to the posterior prostate. $\mu_{s}^{\prime }$ measured by the TRDOT probe showed nearly twofold increase upon coagulation, which is comparable to the 1.4-fold and 1.6-fold increases reported in *ex vivo* prostate samples at 1064 and 633 nm, respectively.^45^

**Table 4:** Reconstructed optical properties at 750 nm (mean ± 1 standard deviation) for native and coagulated canine prostate tissue *in vivo* (n=6 dogs). Measured values are compared to those measured using double integrating sphere measurement in Ref. 45 at 1064 and 633 nm.

	Native		Coagulated	
	$\mu_{a}$ (${\mathrm{mm}}^{-1}$)	$\mu_{s}^{\prime }$ (${\mathrm{mm}}^{-1}$)	$\mu_{a}$ (${\mathrm{mm}}^{-1}$)	$\mu_{s}^{\prime }$ (${\mathrm{mm}}^{-1}$)
750 nm (DR probe)	0.036 ± 0.008	1.2 ± 0.3		
750 nm (TRDOT)	0.034 ± 0.01	0.66 ± 0.2	0.020 ± 0.008	1.1 ± 0.3
1064 nm^45^	0.027 ± 0.003	1.76 ± 0.13	0.019 ± 0.002	2.44 ± 0.14
633 nm^45^	0.073 ± 0.007	2.25 ± 0.05	0.061 ± 0.006	3.59 ± 0.17

**Fig. 7:**
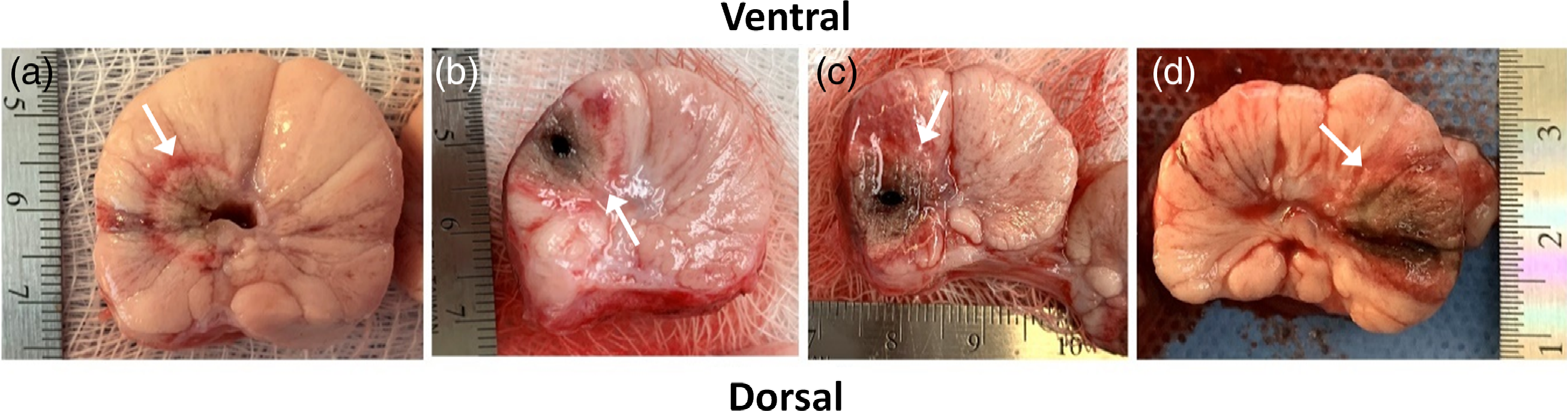
Representative photographs of the photocoagulation zone (indicated by arrows) in four canine prostates excised shortly after completion of the treatment located (a) near the urethra, (b) upper left quandrant, (c) lower left quadrant, and (d) right side. The transrectal probe is located near the dorsal side.

**Fig. 8:**
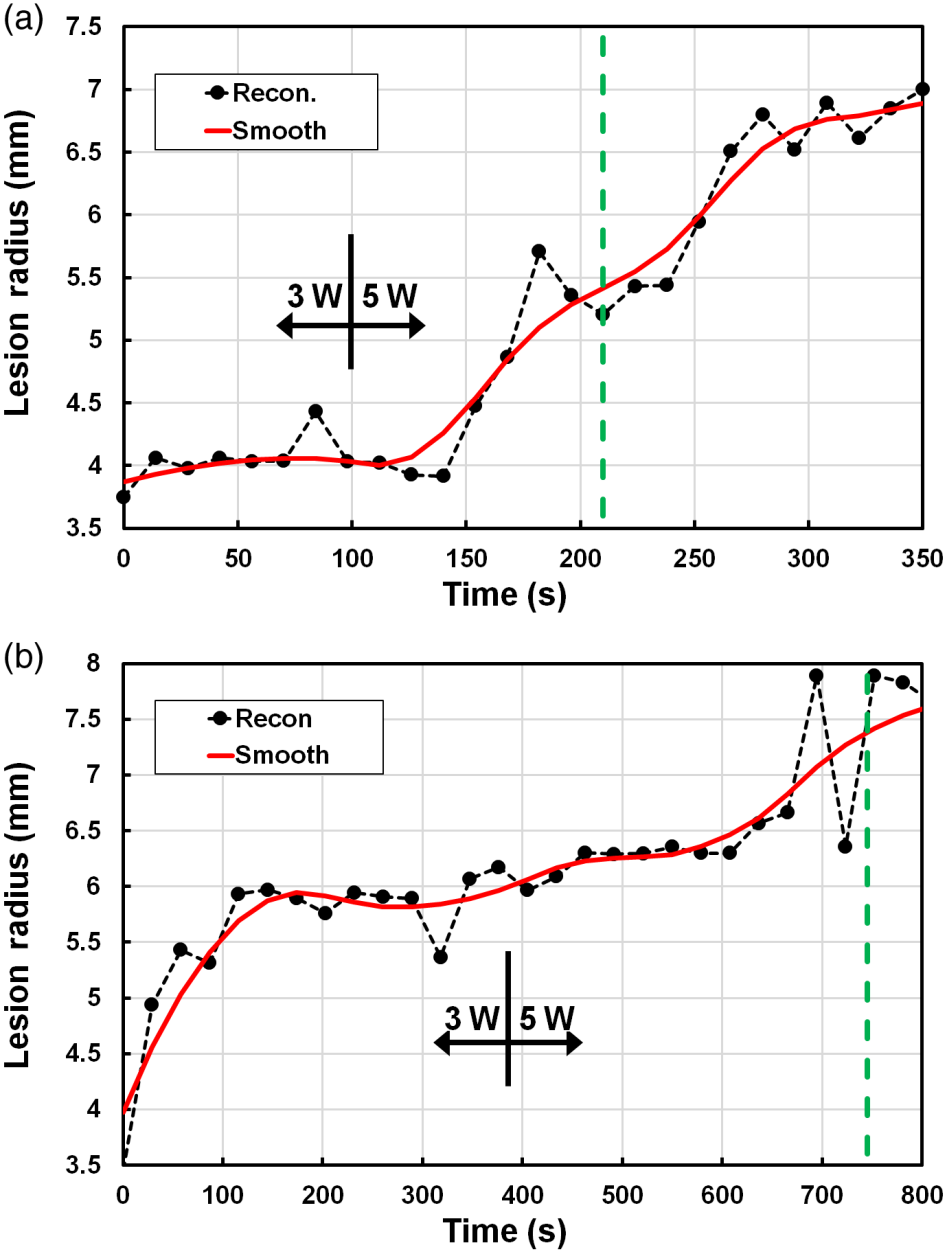
Examples of SBDOT reconstructions during dual-power PTT *in vivo* with (a) four or (b) eight light sources activated with (red) and without (black) 5-point smoothing. PTT starts at t=0  s and stops at the green vertical line.

The interpretation of the tissue response as “binary,” namely coagulated versus unaltered native tissue, is clearly an oversimplification *in vivo*, since temperature-induced homeostatic changes in blood perfusion in the prostate and/or rectum^46^^,^^47^ will affect the optical measurements. Blood accumulation around the PTT fiber (Fig. 7) is a further potential confounding factor that would impact primarily the reconstructed absorption value. The SBDOT reconstructions gave coagulation zone radii between 6 and 12 mm across the 6 animals and converged consistently post-treatment. During PTT, however, the reconstruction is affected markedly also by heat-induced physiological changes within the DOT-interrogated region. In our previous preliminary *in vivo* canine studies, we reported large cyclical fluctuations in TRDOT signals during PTT^27^ and these are apparent also in the examples shown in Fig. S4 in the Supplementary Material. However, the photocoagulation zone expands monotonically and smoothly, suggesting that this behavior is an artefact due to physiological responses to either the heating and/or mechanical stresses from probe placement: it remains to be seen if these fluctuations occur also in human patients during PTT. As shown in Fig. 8, applying five-point smoothing to the reconstructed axial radius largely eliminated the problem, allowing the true coagulation boundary growth to be monitored. This may also be improved further using multiple wavelengths in order to decouple the contribution of changes in blood flow from the true coagulative changes. An additional factor that adversely affects the accuracy of the coagulation monitoring is 28 s TRDOT scan time, during which the photocoagulation radius grows typically by ∼0.5  mm, so that the system is not sampling the same coagulation zone between the start and end of each scan.

Figure 8 presents two specific cases of *in vivo* PTT on canine prostate where the treatment power was increased during PTT. For the case shown in Fig. 8(a), TRDOT used four light sources and all eight detection channels. The post-smoothing reconstruction resulted in a final reconstructed radius of 6.8±0.2  mm compared to 7.0 mm by direct visualization after resection: here the treatment fiber and coagulation front were located at 10 and 3 mm, respectively, from the TRDOT probe surface. Figure 8(b) shows an example using 8 light sources and 8 detection channels that yielded a final reconstructed radius of 7.7±0.2  mm compared to 8.0 mm directly with the PTT fiber and coagulation front at 11 and 3 mm from the TRDOT probe surface.

In both cases, when the treatment laser power was increased at the treatment midpoint, there was a corresponding increased rate of coagulation growth as measured by TRDOT, showing that TRDOT is properly tracking the dynamic changes, as required. We note that, even for closely matched dogs and using normal prostate, there is considerable animal-to-animal variation in the thermal response, which highlights the need for such monitoring in individual cases. Experiments to obtain point-by-point quantitative confirmation versus MR thermometry are planned and are expected to be successful since the final reconstructed size of the coagulation zone is consistent with direct measurements post mortem.

## 4 Conclusions

This work has confirmed the feasibility and accuracy of TRDOT with shape-based reconstruction of the coagulation zone for monitoring PTT treatments. This particular algorithm is not designed for reconstructing the tissue optical properties with high accuracy but, rather, to localize the photocoagulation boundary accurately with respect to the rectal wall. This is the most critical capability to guide treatment so as to achieve complete focal-lesion ablation while ensuring safety. The above experiments in phantoms, *ex vivo* tissues, and an *in vivo* canine prostate model have confirmed that TRDOT can achieve the target accuracy of ±1  mm. Caveats to this conclusion are limitations in the sensitivity and reconstruction accuracy when using our transrectal probe, where data are collected in the reflectance mode and the assumption that the coagulated zone is generally elliptical in shape. Our earlier simulations demonstrated that there were limits in reconstruction accuracy when assuming an elliptical shape in cases where irregularities in the true coagulation zone shape deviated from an elliptical shape by more than 2 mm.^29^

Planned equipment updates aim to reduce the scan time to ∼5  s while also acquiring at multiple wavelengths. Additionally, in the present studies, reconstruction was performed retrospectively using a MATLAB finite-element model for light propagation on a standard PC, typically requiring several minutes. In clinical practice, reconstructions need to be near real time, for which we are exploring alternative reconstruction methods using look-up tables generated from forward Monte Carlo simulations as well as GPU accelerated hardware.^48^ We also plan to combine TRDOT with TRUS imaging that will provide high-resolution anatomical information to complement the functional information from TRDOT, both for improved tumor target localization and PTT treatment monitoring/control. Finally, we note that, although demonstrated here for PTT, TRDOT should be applicable to other thermal focal modalities as well as to treatments in other organs with appropriate DOT probe configurations.
