# Supplementary material for: Preclinical evaluation of a clinical prototype transrectal diffuse optical tomography system for monitoring photothermal therapy of focal prostate cancer
Source: J Biomed Opt. 2022 Feb 1;27(2):026001. doi: 10.1117/1.JBO.27.2.026001 (PMC8806493; doi:10.1117/1.JBO.27.2.026001)
Supplement: Supplementary file 1 [file JBO_027_026001_SD001.pdf]

Supplemental Information

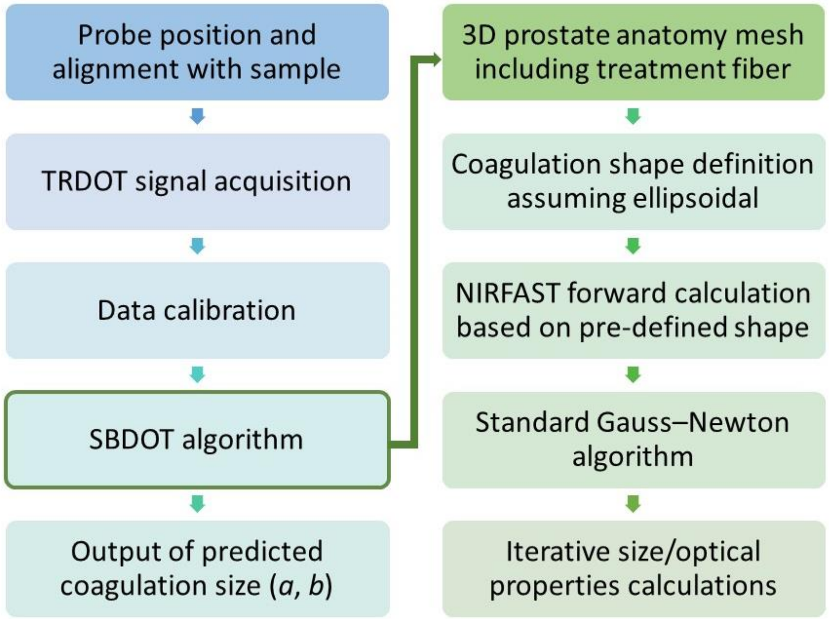

Figure S1. Trans-rectal diffuse optical tomography (TRDOT) data acquisition and shape-based diffuse optical tomography (SBDOT) image reconstruction workflow. Once the TRDOT probe is properly aligned with the expected coagulated zone, signal is acquired by serially sampling each source fiber and collecting simultaneously from each detector fiber. Reference measurements are then made on a calibration phantom of known optical properties and calibration factors for each source-detector pair calculated. These calibration factors are then used to calibrate the sample measurements.

SBDOT reconstruction has been described in detail in Reference 25. A volumetric mesh that includes prostate, rectum, coagulation zone and tissue external to these structures is used in forward light propagation calculations (using NIRFAST). The reconstruction assumes that the coagulated zone is an ellipsoid centered around the cylindrical diffusing fiber used in PTT. Since the treatment fiber location is known, the only optimization parameters are the short and long axis radii of the ellipsoid and the optical properties of the coagulated tissue.

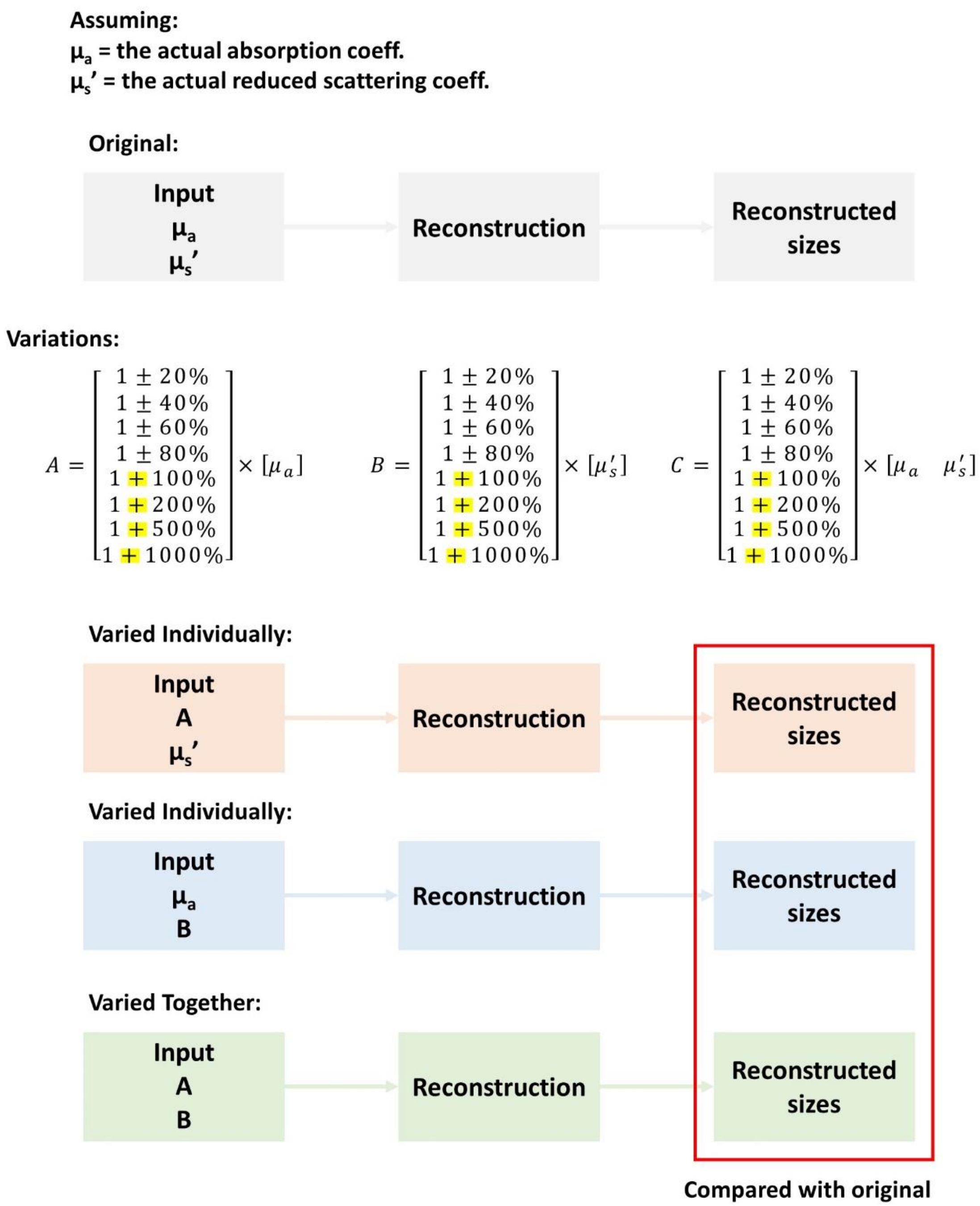

Figure S2. Schematic outlining testing of SBDOT reconstruction to initial estimates of the optical properties. For phantom measurements, the actual optical properties of the simulated coagulation zone were measured using a flat diffuse reflectance probe. SBDOT reconstructions were performed using initial estimates of the coagulated tissue optical properties that varied from the actual properties by increasing or decreasing the optical properties as noted in the figure.

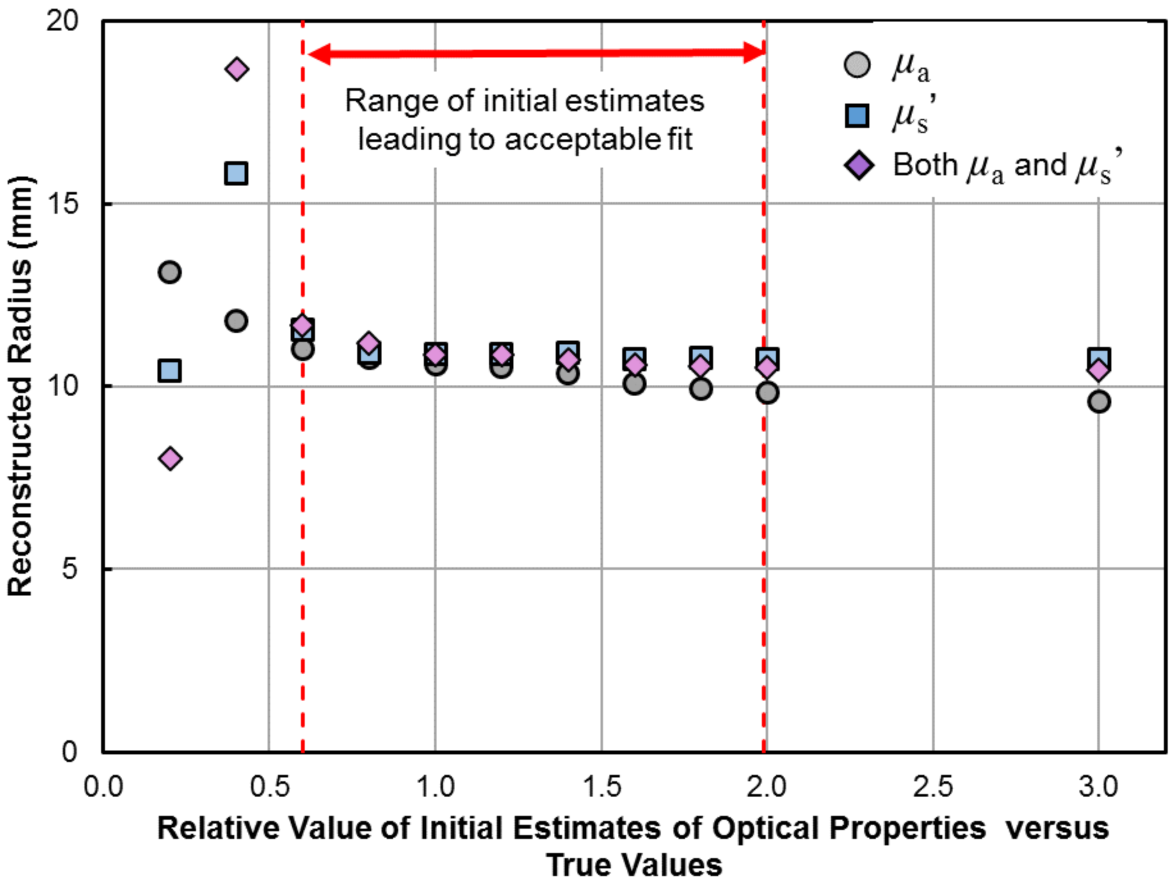

Figure S3: Effect of initial estimates of optical properties on reconstructed coagulation zone radius. Actual lesion radius equals 10.5 mm. Following scheme shown in Fig. S2, reconstructed values are plotted versus relative values of actual optical properties. Initial estimates of the optical properties were separately varied relative to the true value, keeping the other optical property fixed, or both were varied by the same relative amount with respect to the true optical properties. Overestimating the initial optical properties had less impact on the reconstructed lesion size than underestimating the initial optical properties.

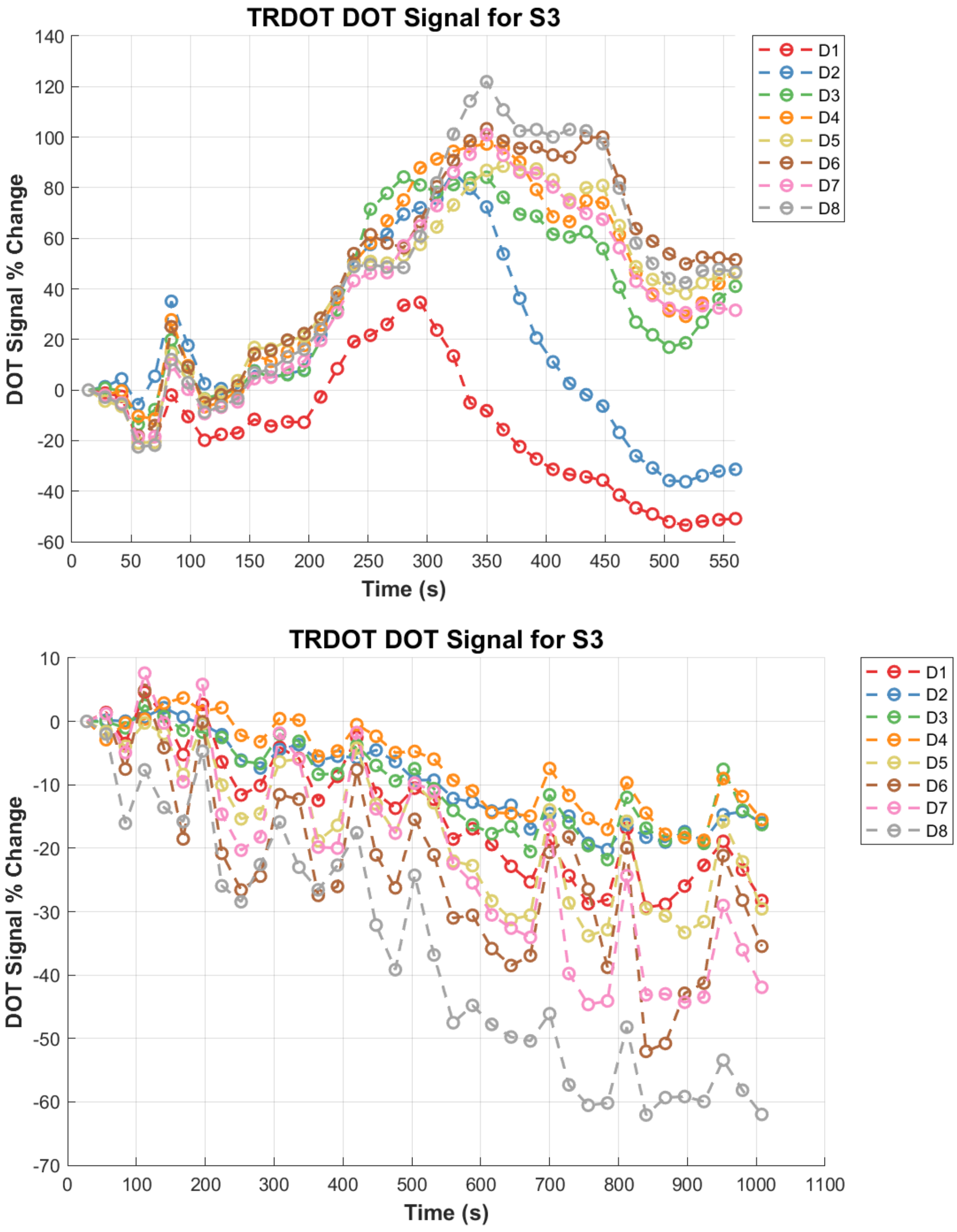

Figure S4. Example of relative changes in optical signal for a single source channel and 8 detector channels during PTT using A) a scanning rate of 1/14 Hz and B) a scanning rate of 1/28 Hz. The source channel, S3, is 3 mm from the center of the optical window on the TRDOT probe. Large modulations in signal are observed in B but not in A.
